# Classification of bruxism based on time-frequency and nonlinear features of single channel EEG

**DOI:** 10.1186/s12903-024-03865-y

**Published:** 2024-01-14

**Authors:** Chunwu Wang, Ajay K. Verma, Bijay Guragain, Xin Xiong, Chunling Liu

**Affiliations:** 1 School of Physics and Electronic Engineering, Hanshan Normal University, Chaozhou, Guangdong 521041 China; 2 School of Electrical Engineering and Computer Science, University of North Dakota, Grand Forks, ND 58202 USA; 3 School of Information Engineering and Automation, Kunming University of Science and Technology, Kunming, 650504 China

**Keywords:** Sleep bruxism, Power spectral density, Fine Tree, Classification, EEG

## Abstract

**Background:**

In the classification of bruxism patients based on electroencephalogram (EEG), feature extraction is essential. The method of using multi-channel EEG fusing electrocardiogram (ECG) and Electromyography (EMG) signal features has been proved to have good performance in bruxism classification, but the classification performance based on single channel EEG signal is still understudied. We investigate the efficacy of single EEG channel in bruxism classification.

**Methods:**

We have extracted time-domain, frequency-domain, and nonlinear features from single EEG channel to classify bruxism. Five common bipolar EEG recordings from 2 bruxism patients and 4 healthy controls during REM sleep were analyzed. The time domain (mean, standard deviation, root mean squared value), frequency domain (absolute, relative and ratios power spectral density (PSD)), and non-linear features (sample entropy) of different EEG frequency bands were analyzed from five EEG channels of each participant. Fine tree algorithm was trained and tested for classifying sleep bruxism with healthy controls using five-fold cross-validation.

**Results:**

Our results demonstrate that the C4P4 EEG channel was most effective for classification of sleep bruxism that yielded 95.59% sensitivity, 98.44% specificity, 97.84% accuracy, and 94.20% positive predictive value (PPV).

**Conclusions:**

Our results illustrate the feasibility of sleep bruxism classification using single EEG channel and provides an experimental foundation for the development of a future portable automatic sleep bruxism detection system.

## Introduction

Bruxism refers to the phenomenon of involuntary contraction of the masticatory muscles under non-physiological conditions, resulting in intermittent masticatory movements [1]. It is divided into awake bruxism and sleep bruxism. Awake bruxism occurs in a conscious state and is usually associated with emotions such as mental tension, anxiety, stress, anger, or depression. However, sleep bruxism occurs at night and is usually caused by sleep apnea or related to sleep disorders. Research have shown that bruxism is common in all age groups and has become an important factor for dental health. It can lead to rapid tooth wear, resulting in pulpitis, narrowing of the occlusal surface, temporomandibular joint disease, nervous system disease, and muscle pain [2]. The prevalence of sleep bruxism in adults ranges from 8 to 16%, whereas in children, it can be as high as 40% [3, 4]. Unfortunately, the etiology of bruxism is complex and the pathogenesis is unclear [5]. Therefore, it is of great significance to detect sleep bruxism as early as possible in order to select the most appropriate treatment method.

Polysomnography (PSG) is considered the gold standard for diagnosing bruxism, but it requires many sensors that increases the complexity causing discomfort to patients. However, electroencephalogram (EEG) provides information related to brain activities that helps to understand relationship between bruxism and brain function. In literature, various physiological signals such as electrocardiogram (ECG), Electromyography (EMG), and EEG have been used in the detection of sleep bruxism. Based on ECG, heart rate variability was used to assess sympathetic cardiac activity in patients with bruxism. Research indicates that patients with bruxism exhibit higher sympathetic cardiac activity compared to healthy controls [6, 7]. Facial EMG can be used to record the potential activity of the patient’s masseter and temporal muscles at night, and the potential value and activity of the EMG can be used to determine whether bruxism occurs [8, 9]. Research has shown that combining ECG and EMG to achieve classification of nocturnal bruxism has also achieved good results [10]. In addition, video capture can also be used to detect the occurrence of bruxism [11], or magnetic resonance imaging for bruxism examination [12]. In addition, EEG is a non-invasive signal, which can be easily obtained from the electrode. It can record neural activity in sleep through different frequency bands. This technology has been widely used as a standard to quantify potential neural activity in sleep research [13, 14]. Researchers have found that most patients with bruxism have significant signs of increased electrical activity in the cerebral cortex during tooth grinding [1]. Sleep bruxism detection has also been conducted by analyzing EEG signals [15–18]. The research of Dakun Lai et al. [10]. shows that the power Spectral density (PSD) of EEG channels in patients with bruxism is significantly higher than that in normal people during rapid eye movement (REM) and awake sleep. Their research also shows that on the basis of the power spectrum characteristics of EEG signals, the fusion of EMG1 and ECG channel signals can achieve better results in the recognition of sleep bruxism patients. Bin Heyat et al. also demonstrate the effectiveness of EEG in detecting sleep bruxism [16]. In addition, the theta activity is also believed to be associated with the occurrence of bruxism [18].

Although the effectiveness of EEG signals has been proven in previous studies, there are problems obtaining EEG signals with too many electrodes causing difficulty in installation and makes patients discomfort. Therefore, identifying the neural correlates of bruxism via single-channel EEG may be of high clinical significance in managing the adverse consequences of sleep bruxism.

The goal of this study is to fully extract the PSD features of EEG in patients with sleep bruxism, thereby attempting to obtain the most effective single channel EEG for identifying bruxism. We propose a new data processing algorithm based on the fusion of multiple EEG frequency band signal features. The algorithm first extracts the PSD values of different EEG frequency bands in REM sleep stage, and then extracts 28 features of these frequency bands in time domain, frequency domain and nonlinear. On the basis of fully extracting the features of EEG signals, the machine learning algorithm is used to identify patients with bruxism. After integrating these features, the classifier can obtain sufficient reference information, providing a reliable basis for the training and accurate classification using machine learning algorithms. The remaining sections of the paper are structured as follows: Section II covers data preparation, data processing, feature extraction, and statistical analysis. Subsequently, machine learning algorithm is applied to classify patients with bruxism. Section III presents the results from data analysis. In Section IV, the research results are thoroughly discussed and compared with existing methods and findings. Section V presents conclusions and prospects for future work.

## Materials & methods

**Fig. 1:**
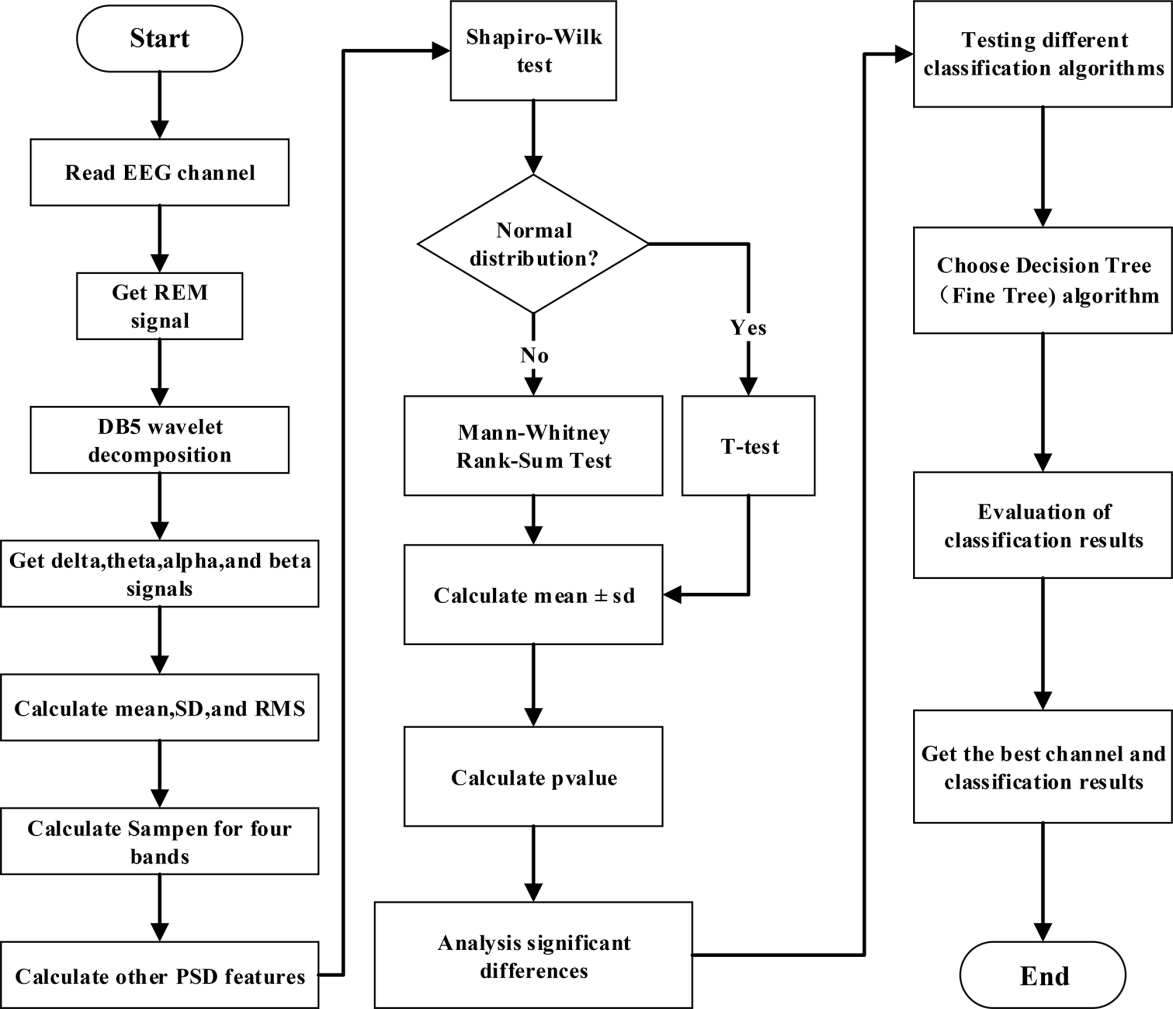
The block diagram of this study

To accurately describe the classification process of bruxism, we designed a data processing flow as shown in Fig. 1. Data processing flow description:

### Data processing and feature extraction

EEG Signal Reading: Read the EEG signals from the REM sleep stages of the subjects. Wavelet Decomposition: Utilize the DB5 wavelet to decompose the EEG signals into delta, theta, alpha, and beta frequency bands. Relative PSD Calculation: Calculate the relative PSD for each frequency band, capturing time-domain, frequency-domain, and non-linear features.

### Statistical analysis

Normality Test: Perform a normality test on the extracted features to ensure their distribution is suitable for statistical analysis. Statistical Test Selection: Determine the appropriate statistical test (e.g., t-test, Mann-Whitney Rank-Sum test) based on the distribution. P-value Calculation and Significance Analysis: Carry out the selected statistical test, calculate the p-value, and determine the significance of the features.

### Classification and result statistics

Classifier Selection: Choose an appropriate classification algorithm based on the features. Classification and Result Collection: Utilize the selected classifier to classify the data and collect the classification results. Optimization and Validation: Iterate over multiple rounds of experimentation and comparison to identify the optimal EEG channel and classify the data effectively.

Through these three key processing steps, we aim to extract significant features from the EEG signals, perform statistical analysis to identify significant features, and utilize these features for effective classification of sleep bruxism. The end result would be the identification of the optimal EEG channel for classification and the resulting classification outcomes.

### Experimental protocol

The data was acquired from cyclic alternating pattern (CAP) sleep database of PhysioNet [19, 20]. It provides representative PSG records of 108 participants with various pathophysiological backgrounds, including 16 controls, 2 bruxism patients, 9 Insomnia, 5 Narcolepsy, 40 Nocturnal frontal lobe epilepsy, 10 Periodic leg movements, 22 REM behavior orders, and 4 Sleep disordered breathing. Each record includes three or more EEG signals, as well as EMG, airflow, respiratory effort, SaO_2_, and ECG signals, and each record has been carefully reviewed by expert neurologists for sleep stage and CAP annotations. The healthy controls who participated in the study exhibited no neurological disorders and were not taking any medications that could affect the central nervous system. All the bipolar EEG channels were sampled at 512 Hz and placed according to 10–20 international electrode placement system [21].

Since the EEG channels collected by each subject in the database are different, we can only select data from 4 health controls (participants: n3, n5, n10, and n11) and 2 bruxism patients (participants: brux1 and brux2) with the same EEG channels (F4C4, C4P4, Fp1F3, F3C3, and C4A1) in this study. According to the annotations made by neurologists, the length of each REM sleep segment was determined to be 30 s. In this study, the total number of REM sleep events is 1295 segments (duration is 38,850 s), including 276 segments of data from bruxism patients. Demographic information for the participants, along with REM sleep events, is presented in Table 1.

**Table 1:** The participant’s demographic information with the period of rem sleep events obtained from hypnogram. (Note: brux stands for bruxism patients and n stands for normal controls. The number at the suffix corresponds to the participant in the Physionet dataset label.)

Participants	Age in Years	Gender	REM Sleep Events	Sleep epoch (sec)	Total time (sec)
brux1	34	M	67	30	2010
brux2	23	M	209	30	6270
n3	35	F	188	30	5640
n5	35	F	232	30	6960
n10	23	M	218	30	6540
n11	28	F	381	30	11,430
	**29.7 ± 5.3**		**1295**	**180**	**38,850**

### Data processing

First, the “edfread” function is used to read each polysomnographic record, which helps to obtain the identification and specific data of each channel, providing a foundation for subsequent analysis. Then, the “ScoringReader” function is executed to obtain the identification code and duration of each sleep stage. This information is crucial for accurately determining an individual’s sleep state. Finally, EEG data from the REM sleep stage are extracted, which prepare for further in-depth research. After preparing the REM segment of the EEG signal, the next step is to calculate its PSD. First, the REM segment of the EEG signal is decomposed into different frequency bands using the DB5 wavelet, including (δ, 1–4 Hz), theta (θ, 4–8 Hz), alpha (α, 8–13 Hz), and beta (β, 13–30 Hz). Next, the PSD was calculated using Welch estimate with Hamming window size of 128 samples with 50% overlap and 256 discrete Fourier transform points for each frequency bands. Finally, the absolute PSD (APSD) were calculated.

1 $$RPS{D_n} = \frac{{PS{D_n}}}{{PS{D_\delta } + PS{D_\theta } + PS{D_\alpha } + PS{D_\beta }}} \times 100\% \,,\,(n \in [\delta ,\theta ,\alpha ,\beta ])$$

The relative PSD on each frequency band was obtained from the ratio of absolute power of each four frequency bands to the total power within the spectrum of 1–30 Hz [13]. The delta band is chosen beyond 1 Hz to minimize low frequency head movement and ocular artifacts below 1 Hz. The PSD analysis within the frequency band 1–30 Hz is chosen since EEG information relating to sleep relies within this spectrum.

In order to conduct subsequent statistical analysis and obtain significant feature indicators for characterizing bruxism, we performed subsequent processing. Various features were extracted by time domain, frequency domain, and sample entropy (SampEn) for each REM sleep epochs from healthy controls and bruxism patients. For time domain features, Mean value, Standard Deviation (SD), and Root Mean Square (RMS) were calculated for each of the frequency bands [22]. For frequency domain features, the relative power spectral density (RPSD) including RPSD (δ), RPSD (θ), RPSD (α), RPSD (β), and ratios including (θ + α)/β, α/δ, α/θ, and α/β were computed as the additional features [23]. For nonlinear features, the Shannon entropy (SampEn) was calculated with default parameters (the maximum template length is 5 and the matching threshold is 0.2). Finally, a total of 28 features including 12 time domain features, 12 frequency domain features, and 4 non-linear analysis features were extracted.

### Statistical analysis

The statistical analysis is performed to compare differences in EEG features from five EEG bipolar electrodes (channels) among healthy controls and bruxism patients. A Shapiro-Wilk test is used to examine the normality of data and a suitable parametric or non-parametric test is adopted based on the data distribution [24]. Since the data samples are independent, either two sample t-test as a parametric test for normalized data or Mann-Whitney Rank-Sum test as a non-parametric test for non-normalized data can be performed to obtain statistical comparison among two groups: healthy controls and bruxism patients.

### Classification and cross-validation

The systematic empirical evaluation of various machine learning algorithms shows that Decision tree (Fine Tree) algorithm is the optimal algorithm for the classification of bruxism in CAP database. Therefore, unless stated otherwise, all the data discussed in this paper is the output of the Fine Tree classifier.

Decision tree is a basic method of classification and regression. It achieves classification by dividing input features into different subsets layer by layer. The core idea of decision tree classifier is to determine the decision rules for classification by systematically dividing features, thus facilitating data classification. Due to its resemblance to the branches of a tree, this decision graph is called a decision tree. During the decision tree classification process, instances are segmented based on features and assigned to distinct categories. The main advantages of this method are model readability, easy to understand, fast classification, fast modeling, and prediction [25].

## Result

**Fig. 2:**
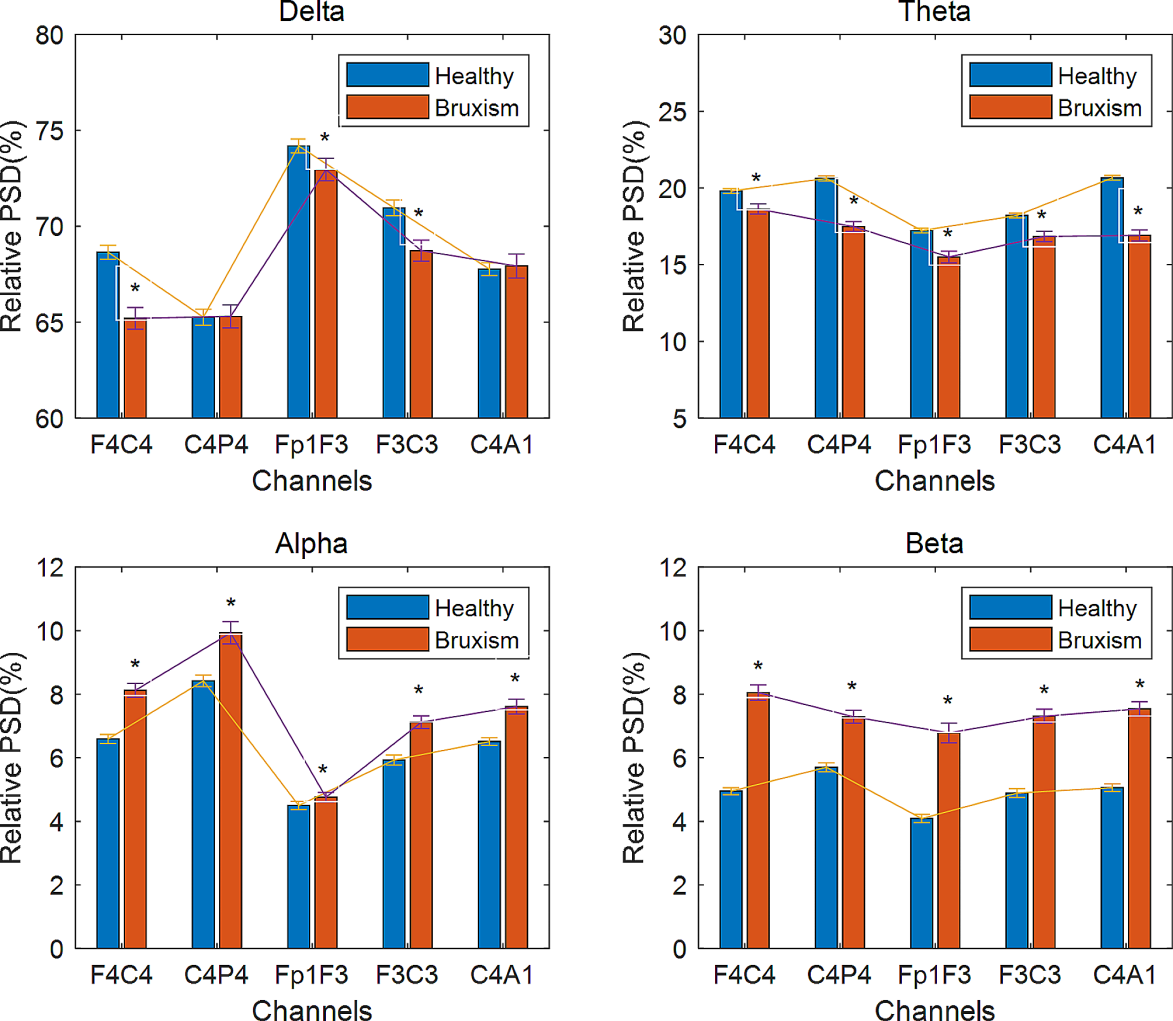
The relative spectral power distributed (mean ± SE) in four EEG frequency bands between two groups: healthy controls and bruxism patients with five EEG bipolar channels: F4C4, C4P4, Fp1F3, F3C3, and C4A1 during REM sleep. The ‘*’ represents alpha levels: *p* < 0.05 correspond to significant differences

The normality test in the data have exhibited mixed behavior and the samples are independent between the two groups. Therefore, Mann-Whitney Rank-Sum test is used to test the significant difference. The significance level is set at the alpha criterion α = 0.05. The relative PSD in five EEG channels during REM sleep stage among healthy controls and bruxism patients are compared and their trends (mean ± SE) are illustrated in Fig. 2.

The statistical analysis comparing healthy controls and bruxism patients revealed a significantly lower relative delta power (*p* < 0.05) in three channels (F4C4, Fp1F3, and F3C3), as well as a significant decrease in relative theta power across all five channels. In contrast, the relative alpha and beta power exhibited a significant increase (*p* < 0.05) in all five channels for bruxism patients. The overall results depicted decrease in low frequency band power (delta and theta bands) and increase in high frequency band power (alpha and beta bands) for bruxism patients. In low frequency bands, the profound lower relative power was observed in theta band and more dominant in C4A1 and C4P4 channels whereas, the profound higher in relative power was observed in beta band for high frequency bands and more dominant in Fp1F3 and F4C4 channels. Among four frequency bands, relative beta power had most significant differences for sleep bruxism patients.

The significance of 28 features from each channel was compared between healthy controls and individuals with bruxism. As an instance, Tables 2, 3, 4 and 5 compare all the features of participants under healthy controls and bruxsim for C4P4 channel. The Shapiro-Wilk test was used to observe the normality of data distribution and the result showed mixed behavior due to limited sample size. Therefore, Wilcoxon rank sum test was used for statistical analysis [22]. The test result at *p* ≤ 0.05 was considered significant. The results showed that SD (α), SD (β), RMS (α), RMS (β), SampEn (α), SampEn (β), RPSD (δ), RPSD (θ), RPSD (α), (θ + α)/β, α/δ, α/θ, α/β, and APSD (α) had significant differences between healthy controls and bruxism patients, demonstrating that these features are effective for classification. Most of the features used for the classification task were found to be significant.

**Table 2:** The comparison of p-values from 6 participants with 12 time domain features under two psychological states (healthy controls and bruxsim) for C4P4 channel

SN	Features	Healthy controls (mean ± SD) (mV)	Bruxism (mean ± SD) (mV)	p-value
1	Mean(δ)	0.018 ± 0.006	0.064 ± 0.045	0.566
2	Mean(θ)	-1.021E-5 ± 1.092E-5	-2.336E-±5.055E-5	0.989
3	Mean(α)	2.955E-6 ± 1.991E-6	-2.198E-6 ± 1.238E-5	0.842
4	Mean(β)	1.106E-6 ± 7.875E-7	5.211E-7 ± 5.211E-6	0.959
5	SD(δ)	8.280 ± 0.233	6.976 ± 0.708	<=0.001**
6	SD(θ)	0.944 ± 0.009	1.576 ± 0.063	<=0.001**
7	SD(α)	0.309 ± 0.005	0.835 ± 0.033	<=0.001**
8	SD(β)	0.135 ± 0.004	0.608 ± 0.052	<=0.001**
9	RMS(δ)	8.281 ± 0.233	6.981 ± 0.710	<=0.001**
10	RMS(θ)	0.944 ± 0.009	1.576 ± 0.063	<=0.001**
11	RMS(α)	0.309 ± 0.005	0.835 ± 0.033	<=0.001**
12	RMS(β)	0.135 ± 0.004	0.608 ± 0.052	<=0.001**

The ‘*’ and ‘**’ represent alpha levels: *p* = 0.05, and *p* = 0.001 correspond to significant differences, respectively

**Table 3:** The comparison of p-values for 6 participants in frequency domain features in (RPSD and ratios) under two psychological states (healthy controls and bruxsim) for C4P4 channel

SN	Features	Healthy controls (mean ± SD)(%)	Bruxsim (mean ± SD)(%)	p-value
1	RPSD(δ)	65.26 ± 0.422	65.302 ± 0.597	0.677
2	RPSD(θ)	20.619 ± 0.158	17.480 ± 0.317	<=0.001**
3	RPSD(α)	8.424 ± 0.185	9.931 ± 0.348	<=0.001**
4	RPSD(β)	5.696 ± 0.140	7.287 ± 0.197	<=0.001**
5	(θ + α)/β	8.246 ± 0.289	4.721 ± 0.199	<=0.001**
6	α/δ	0.159 ± 0.005	0.169 ± 0.009	<=0.001**
7	α/θ	0.382 ± 0.008	0.633 ± 0.029	<=0.001**
8	α/β	1.601 ± 0.017	1.434 ± 0.032	<=0.001**

The ‘*’ and ‘**’ represent alpha levels: *p* = 0.05, and *p* = 0.001 correspond to significant differences, respectively

**Table 4:** The comparison of p-values for 6 participants in frequency domain features (APSD) under two psychological states (healthy controls and bruxsim) for C4P4 channel

SN	Features	healthy controls (mean ± SD) (mV2/Hz)	Bruxsim (mean ± SD) (mV2/Hz)	p-value
1	APSD(δ)	83.048 ± 16.083	129.720 ± 65.344	<=0.001**
2	APSD(θ)	16.698 ± 2.191	17.517 ± 5.569	<=0.001**
3	APSD(α)	3.885 ± 0.111	7.135 ± 2.170	<=0.001**
4	APSD(β)	2.367 ± 0.059	4.292 ± 1.203	0.188

The ‘*’ and ‘**’ represent alpha levels: *p* = 0.05, and *p* = 0.001 correspond to significant differences, respectively

**Table 5:** The comparison of p-values for 6 participants with 4 sample entropy features under two psychological states (healthy controls and bruxsim) for C4P4 channel

SN	Features	healthy controls (mean ± SD)	Bruxsim (mean ± SD)	p-value
1	SampEn(δ)	0.530 ± 0.002	0.572 ± 0.006	<=0.001**
2	SampEn(θ)	0.822 ± 0.001	0.810 ± 0.005	0.695
3	SampEn(α)	0.977 ± 0.003	0.957 ± 0.007	0.01*
4	SampEn(β)	1.378 ± 0.006	1.303 ± 0.013	<=0.001**

The ‘*’ and ‘**’ represent alpha levels: *p* = 0.05, and *p* = 0.001 correspond to significant differences, respectively

**Fig. 3:**
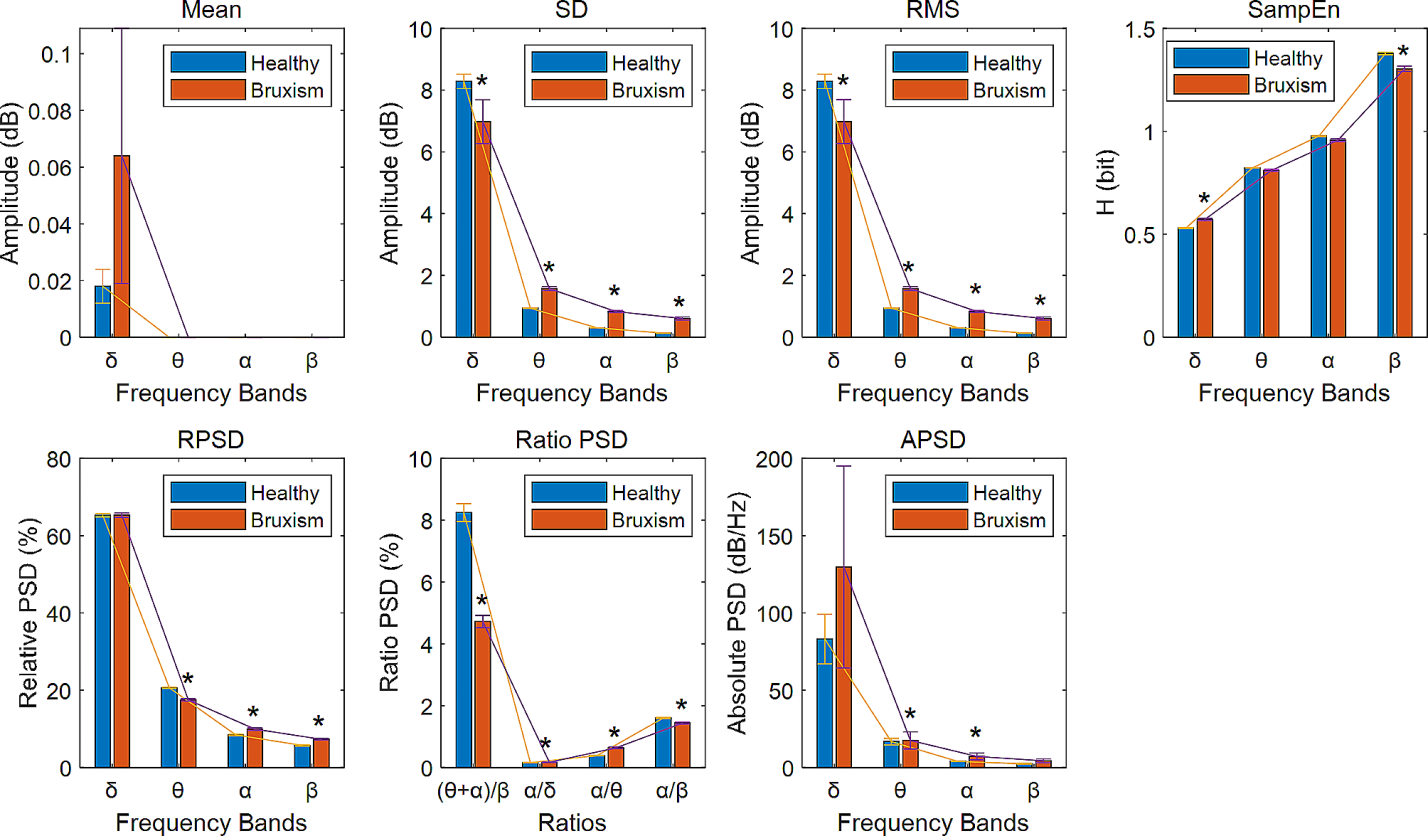
The 28 features distributed (mean ± SD) in four EEG frequency bands: δ, θ, α, and β between two groups: healthy controls and bruxism patients with channel C4P4 during REM sleep. ‘*’ represent the alpha level (*p* < 0.05) for significant difference

To visually illustrate the behavior of each feature across different channels, Fig. 3 displays all 28 features of participants from both healthy controls and bruxism patients for the C4P4 channel, as an example. Upon observing Fig. 3, it can be inferred that, in comparison to other frequency bands, the delta frequency band exhibits significant amplitude values on all features, except for a smaller amplitude on the SampEn feature. There were significant differences in SD, RMS, SampEn and four ratio PSD characteristics between healthy controls and bruxism patients. Features related to the theta, alpha, and beta frequency bands exhibited distinct behavioral patterns, with some of them also demonstrating statistical significance.

All the above mentioned 28 features (see Tables 2, 3, 4 and 5 for detail) can provide basis for correct identification of bruxism patients from different aspects. By utilizing these features, we evaluated various machine learning algorithms and ultimately concluded that Fine Tree is the optimal algorithm for bruxism classification in the CAP database. Fine Tree classifier with default parameters, in MATLAB (Mathworks Inc., MA, USA), was used for classification. Table 6 lists the sensitivity, specificity, accuracy, and positive predictive value (PPV) obtained from all the five channels in the parietal region using five-folds cross validation. It showed the C4P4 channel achieves the highest classification performance with sensitivity (95.59%), specificity (98.44%), accuracy (97.84%), and PPV (94.20%) in C4P4 channel.

**Table 6:** Comparison of bruxism classification with five-folds cross-validation for respective EEG channels

Channel	Sensitivity (%)	Specificity (%)	Accuracy (%)	PPV(%)
F4C4	86.11	97.22	94.75	89.86
**C4P4**	**95.59**	**98.44**	**97.84**	**94.20**
Fp1F3	91.70	96.80	95.75	88.04
F3C3	91.82	97.17	96.06	89.49
C4A1	88.64	96.67	94.98	87.68

## Discussion

The purpose of this study was to comprehensively investigate the utility of single EEG channel for classification of bruxism patients from healthy controls in REM sleep. Literature relies primarily on PSD changes in the EEG frequency bands, including delta, theta, alpha, and beta to estimate the characteristics of neural activities in the brain [7, 10, 15]. However, in this study, in order to obtain sufficient signal features, we combined time domain, frequency domain, and nonlinear features from the EEG signals.

The artifacts in EEG signals are highly complex and can appear in any frequency bands. The effective identification and filtering of artifacts such as swallowing, yawning, jaw alignment, snoring, or apnea is challenging that requires further in-depth research. However, in our database, the recordings were annotated by expert neurologists with information on sleep stages (wakefulness, S1-S4 sleep stages, REM, and body movements), body position (left, right, prone, or supine), and duration (seconds). Among all these annotated EEG signals, we only extracted the REM segment of interest, and this data segment already excluded body movements and body positions. Further, to remove slow frequency motion artifacts, we used DB5 wavelet transform to decompose the single-channel EEG signals into multi-frequency bands and then obtained the EEG signals at selected frequency bands within 1–30 Hz. This further filter out slow frequency artifacts below 1 Hz and higher frequency artifacts above 30 Hz.

Since EEG signals can directly reflect the neural activities of the human brain and more directly reflect the influence and characteristics of bruxism at the neural level, it is of great importance in the analysis of bruxism. In the present study, we used statistical methods to analyze EEG frequency bands to compare the PSD dynamics from EEG among bruxism patients and healthy controls during REM sleep. Since there are many EEG channels, it will be beneficial if the channels and frequency bands reflecting the occurrence of bruxism are selective that simplifies the design of EEG acquisition instrument having improved classification accuracy. Therefore, in this paper, we analyzed five different EEG channels and revealed the most effective frequency band of EEG channel in characterizing sleep bruxism.

Tang Jin Cheng et al. [26] made important achievements in the research of brain-computer interface by extracting the mean, standard deviation, root mean square value and other time-domain features of EEG signals, revealing that these time-domain features are helpful to understand the data distribution characteristics of different EEG frequency bands. By conducting experiments on multiple channels of EEG signals, it was found that the standard deviation and variance features were the most significant [26]. Hayat [16] and Lai [10] analyzed the average, maximum, and minimum values of the average normalized power spectrum using two EEG channels, revealing the effectiveness of power spectrum in the detection of sleep bruxism. Among time-domain features, mean, SD, and RMS were typical approaches to measure the amplitude of EEG [27–29]. Therefore, we also attempted to extract the relevant time-domain features of EEG signals and evaluate their role in the identification of bruxism. In this study, we conducted statistical analysis on the time-domain features of PSD (mean, SD, and RMS) in each frequency bands of EEG and found that SD(δ), SD(θ), SD(α), SD(β), RMS(δ), RMS(θ), RMS(α), and RMS(β) had significant differences (*p <* 0.001) between bruxism patients and healthy controls.

The frequency domain features can represent the proportion of components in different frequency bands, thus representing the activity of cranial nerves [30, 31]. Studies have also found that some ratios of different bands, which includes following derived indices (θ + α)/β and α/β [32], and (θ + α)/(α + β) and θ/β [23] are useful for EEG features analysis. In this study, the RPSD including RPSD (δ), RPSD (θ), RPSD (α), RPSD (β), and ratios including (θ + α)/β, α/δ, α/θ, and α/β were computed. We found that many frequency domain features have significant differences (*p <* 0.001) that includes RPSD(θ), RPSD(α), RPSD(β), α/δ, α/θ, α/β, APSD(δ), APSD(θ), and APSD(α).

The nonlinear feature (Shannon entropy, SampEn) is a measure of the complexity of a system, and it is very effective for the analysis of short-length time series. The SampEn of EEG signal can be used to reveal the potential regularity and periodicity of data, which has been proven to accurately distinguish patients diagnosed with depression from the control group, which can serve as a highly sensitive and clinically relevant marker [33]. Mahshid Dastgoshadeh et al. also showed that the SampEn feature of EEG signals is a good tool for detecting epilepsy [34]. Richman et al. have revealed that SampEn is more suitable for the study of biological time series signals [35]. In this study, we found that the behavior of SampEn (δ), and SampEn (β) had significant differences (*p <* 0.001) and SampEn(α) also had significant differences (*p <* 0.05) between bruxism patients and healthy controls.

In order to maximize the extraction of EEG frequency band features, we extracted 28 time-domain, frequency-domain, and nonlinear features (SampEn). Then statistical methods were used to compare the ability of each feature in classifying bruxism. The statistical results show that most of the features are effective to identify bruxism patients from healthy controls, especially in the high frequency bands (theta, alpha, and beta) that vary significantly. This variation in the high frequency bands might be due to the activation of neural activities involved in clenching teeth [1]. Mastication is controlled by central nervous system of brain [5, 36] and the cause of involuntary clenching during sleep bruxism is mostly unknown. The relative increase in high power around the frontal and central EEG channels in our results indicates high-frequency neural oscillations in the fronto-central brain regions, contributing to involuntary teeth grinding during sleep bruxism. Certainly, further analysis with larger sample size needs to be performed to support the claim with higher confidence level.

Table 7 shows the comparison between the classification performance of this study and previous studies. In the literature, among the detection methods of bruxism patients, EMG, ECG and EEG have been used by researchers. The accuracy of identifying bruxism patient based on EMG can reach 82.8% [9]. However, after combining EEG, EMG and ECG signals, the bruxism recognition accuracy reached 97.21% [10]. This method needs to collect EEG, EMG and ECG signals at the same time that increases the difficulty in signal acquisition and signal processing, and is not suitable for using portable devices to identify bruxism patients. Therefore, researchers began to study the strategy of only using EEG to identify bruxism, but the recognition rate of bruxism was not ideal. When utilizing only time-domain features (maximum, minimum, and mean) of PSD, even with the fusion of features from two EEG channels, the accuracy of bruxism classification can only achieve 81.25% [16]. Due to the correlation between EEG channels, even using channel fusion does not effectively improve classification accuracy. However, if the features of a single channel EEG signal can be fully extracted, it may help to improve classification accuracy. Based on this assumption, we used the same database as in literature [10, 16, 17] for our study. Our experimental results showed that when using 28 time domain, frequency domain, and nonlinear features, we could achieve higher accuracy (97.84%) from single EEG channel (C4P4). Our results demonstrated bruxism detection using single channel EEG.

**Table 7:** Comparison classification performance between the proposed and previous works

Authors	Signal	Method	Channel	Sleep stage	Accuracy (%)
E. O’Hare et al. [9]	EMG	Linear discriminant analysis	EMG	Awake	82.8%
Bin Heyat et al. [16]	EEG	Decision tree	C4P4,C4A1	REM	81.25%
Bin Heyat et al. [17]	EEG,EMG,ECG	Hybrid Machine Learning Classifier	ECG1,ECG2,C4P4,C4A1	REM	97%
D. Lai et al. [10]	EEG,EMG,ECG	Decision tree	EEG,EMG,ECG1	REM	97.21%
**Present**	**EEG**	**Decision tree (Fine Tree classifier)**	**C4P4**	**REM**	**97.84%**

## Conclusion

While previous studies have put several schemes for bruxism recognition, the utilization of multi-channel data acquisition has rendered the detection system more complex and unsuitable for portable devices. Some studies still have insufficient feature extraction and low recognition rates. Our research findings will help explore the potential of designing a portable sleep bruxism detection system based on a single channel EEG. We propose a classification method for sleep bruxism using a single EEG channel combined with time domain, frequency domain, and nonlinear features. Experimental results showed that there are significant differences (*p* < 0.05) in all the 28 features except for Mean(δ), Mean(θ), Mean(α), Mean(β), RPSD(δ), APSD(β), SampEn(θ) between bruxism patients and healthy controls. Investigation on classification had also confirmed that these features were useful for classification.

Multi-channel EEG devices are inconvenient to place electrodes, while using a single channel EEG acquisition can effectively reduce the complexity of the detection device, facilitate the installation of the device, and reduce the discomfort caused by data acquisition. The results of our study also showed that the classification performance of different channels of the brain were different. Among the channels, the C4P4 channel had the best classification results (the accuracy can reach 97.84%). In the 10–20 international electrode placement system, C4P4 is located on the right side of the Parietal and Central of scalp. Our experimental results suggest that placing electrode in this area is conducive to the development of a single channel portable bruxism detection system.

Admittedly, our sample size is limited to 2 bruxism patients and 4 healthy controls. A larger sample size and more uniform population distribution (age, sex, and body weight) will give people more confidence in extending the results to the prediction and monitoring of patients with bruxism. Although the CAP sleep database of PhysioNet only has fewer patients with bruxism, it has a long record time for each subject, and expert neurologists annotate the data, making it authoritative. The database has important academic research value and has been used by multiple groups for research on bruxism. In addition, EEG has been used to characterize sleep bruxism in this study, we have extracted more effective features, and achieved higher classification performance using single channel EEG. This finding not only further supports the previously reported findings, but also extends the ability of single channel EEG to recognize bruxism.

Indeed, the classification of bruxism in clinical research is a continuously evolving process that requires the combination of various assessment methods to obtain more reliable results. Simply relying on database analysis is not sufficient for accurate classification of bruxism. Attempts have been made in the literature to use EMG, ECG, or EEG signals to characterize the features of bruxism occurrence [9, 10, 15]– [18]. Although these methods cannot fully replace the accurate judgments of doctors, they can serve as medical auxiliary equipment to provide monitoring and warnings to potential bruxism patients or assist dentists in diagnosis. These data and analysis methods still have certain reference value for the diagnosis of “bruxism”.

Additionally, we also need to clarify that the proposed method is only suitable for sleep bruxism recognition and is not a general bruxism classification system. Therefore, it is very important to comprehensively consider various data and analysis methods in the research and diagnosis of bruxism to improve the accuracy and reliability of classification. In the future, additional physiological signals such as ECG, EMG, and SaO_2_ will be analyzed across all five sleep stages. The study will also be expanded to assess the recognition ability of bruxism in different sleep stages.

## Data Availability

The data that support the findings of this study are available from PhysioNet [19, 20], which are publicly available. PhysioNet is a repository of freely available medical research data, managed by the MIT Laboratory.

## Availability of data and material

All data generated or analyzed during this case are included in this published article.
